# Relationships between ENDS-Related Familial Factors and Oral Health among Adolescents in the United States

**DOI:** 10.3390/healthcare10020402

**Published:** 2022-02-21

**Authors:** Man Hung, Martin S. Lipsky, Amir Mohajeri, Clarissa Goh, Jungweon Park, Chase Hardy, Sharon Su, Frank W. Licari

**Affiliations:** 1 College of Dental Medicine, Roseman University of Health Sciences, 10894 S. River Front Parkway, South Jordan, UT 84905, USA; mlipsky@roseman.edu (M.S.L.); amohajeri@roseman.edu (A.M.); jpark6@student.roseman.edu (J.P.); chardy@student.roseman.edu (C.H.); ssu@student.roseman.edu (S.S.); flicari@roseman.edu (F.W.L.); 2 Riverton High School, Riverton, UT 84065, USA; clarissagoh4@gmail.com

**Keywords:** eectronic nicotine delivery dystems, familial factors, social factors, oral health, dental research

## Abstract

The influence of familial and social environments plays a significant role in Electronic Nicotine Delivery System (ENDS) use and may contribute to poor oral health among adolescents. This study utilized the Population Assessment of Tobacco and Health (PATH) database and included youths aged 12 to 17 years who reported no history of dental health issues at baseline. Adjusted odds ratios (AOR) were used to examine the association between END-related familial factors and oral health among adolescents in the United States, with statistical significance set at *p* < 0.05. The sample consisted of 3892 adolescents (weighted N = 22,689,793). Parents’ extremely negative reaction towards ENDS when they found their children using ENDS (AOR = 0.309) was connected to a lower risk of oral health issues. The findings suggest that clinicians and policymakers need to consider the roles of these factors when developing strategies to improve oral health outcomes.

## 1. Introduction

The past few decades have seen a marked decrease in cigarette use in the United States [1]. However, this decline has slowed, and some experts now predict an increase in smoking fueled by the increasing popularity of alternative tobacco products [2]. The most prevalent of these new products are electronic nicotine delivery systems (ENDS) [3] which are battery-operated devices that substitute tobacco with the aerosolization, or “vaping” of a flavored solution usually containing nicotine. Originally touted as smoking cessation devices, ENDS manufacturers use flavored solutions and sleek, attractive designs to attract young users who rarely use these products for smoking cessation [4,5]. From 2019 to 2020, disposable e-cigarette use increased 1000% among high school students and 400% among middle school students [6]. In 2021, the annual National Youth Tobacco Survey found that 2.06 million US adolescents used e-cigarettes [7], making them the most commonly used smoking product by adolescents [8,9,10]. The rise in ENDS use raises two key concerns, their potential harms and their connection to future combustible tobacco use.

Although marketed as less harmful than combustible tobacco products, research indicates that ENDS use adversely affects health. ENDS solutions contain toxic compounds such as lead and nickel [11] and most contain nicotine [12]. Highly addictive, nicotine plays a role in the pathogenesis of several diseases including neurodegenerative disorders [13], cardiopulmonary diseases [14], and cancer [15,16,17]. Often overlooked is their impact on oral health. ENDS use increases the risk of dental disease [18,19] by triggering the release of pro-inflammatory cytokines in the oral cavity [20], damaging gingival fibroblasts [21] and leaving a residue on oral tissues that promotes dental decay [22]. Over time, ENDS use can also cause endothelial dysfunction [23,24] which is associated with periodontal disease [25,26]. These mechanisms and a 2019 cross-sectional study that found ENDS users experience worse oral health than non-users [27] support a connection between ENDS use and poor oral health.

Evidence implicates familial factors as strong predictors of smoking uptake among adolescents. Having parents [28], siblings [29], or other family members [30,31] who smoke significantly increases the likelihood of adolescents smoking. Adolescents are less likely to smoke if they believe that their parents would be upset or respond negatively [32], and anti-smoking beliefs are linked to having a smoking-prohibitive family [33]. Furthermore, family norms toward smoking were consistently found to be more impactful than less direct social influences [34]. 

Although research links familial smoking behaviors and attitudes with smoking [35] and ENDS use with poorer oral health [36], no study has directly explored the influence of ENDS-related familial factors on oral health. This study sought to explore what associations, if any, exist between ENDS-related familial factors and oral health in adolescents. Identified associations should be valuable for helping dentists, other healthcare providers, and policymakers to develop and test strategies to improve oral health outcomes among adolescents.

## 2. Materials and Methods

The Population Assessment of Tobacco and Health (PATH) study is a nationally representative longitudinal study of 45,971 United States adults (18 years and older) and youth (12–17 years). PATH collects self-reported information on tobacco-use patterns and associated health behaviors through audio computer-assisted self-interviews to examine tobacco use. PATH is an ongoing longitudinal study, and its data collection began with Wave 1 in 2013, with five subsequent data collections for youth participants from Wave 1 to Wave 5. All wave 1 respondents stayed eligible for follow-up interviews if they remained residents of the US and were not incarcerated.

PATH uses sampling weights to develop population estimates representative of the noninstitutionalized United States population. Youths who participated in three waves of the PATH study (19 October 2015–23 October 2016 (Wave 3), 1 December 2016–3 January 2018 (Wave 4), 1 December 2018–30 November 2019 (Wave 5)) were eligible for the present study. Details regarding PATH survey interview procedures can be found at http://doi.org/10.3886/Series606 (Accessed on 16 January 2022).

### 2.1. Inclusion and Exclusion Criteria

**Figure 1:**
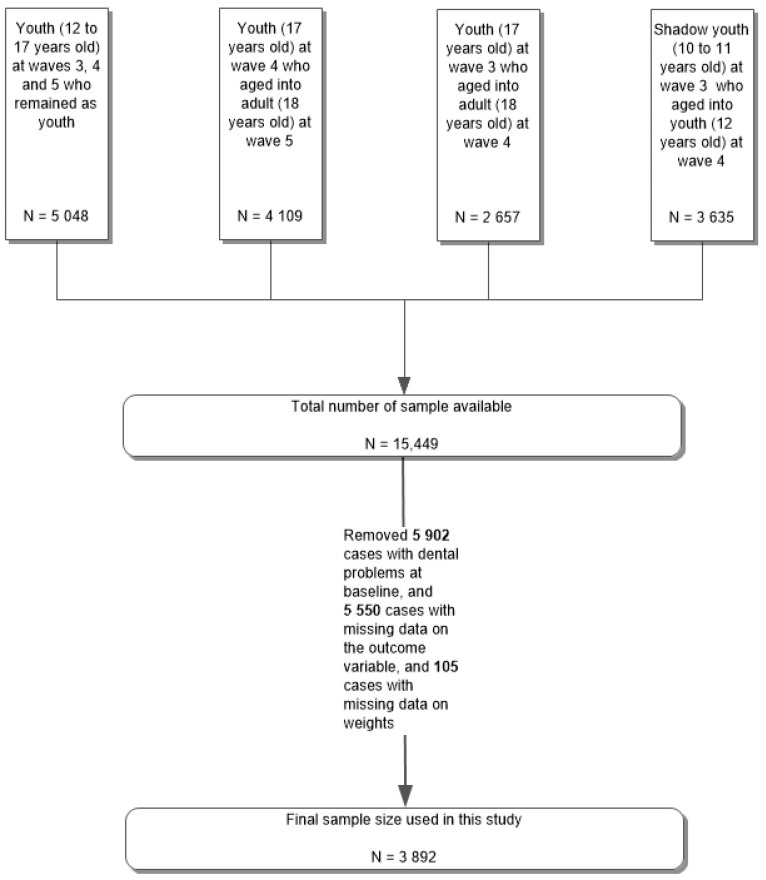
Flowchart displaying sample selection based on inclusion and exclusion criteria.

Since the variables from waves 1 and 2 were different than those from waves 3, 4 and 5, this study excluded waves 1 and 2’s data. The inclusion criteria for sample selection were: (1) Youths who were 12 to 17 years old in all three waves (3, 4, 5); (2) Youths who were 17 years old at wave 4 and aged into adults (18 years old) at wave 5; (3) Youths who were 17 years old at wave 3 and aged into adults (18 years old) at wave 4; and (4) Shadow youths who were 10 to 11 years old at wave 3 and aged into youths (12 years old) at wave 4. This yielded an eligible sample of 15,449 individuals prior to applying exclusion criteria. The exclusion criteria were: (1) Individuals with dental problems at baseline; (2) Individuals with missing data for the outcome variable; and (3) Individuals with missing data for the weight variable. After applying the exclusion criteria, the final sample size for this study was 3892. Figure 1 displays the sample selection process based on the stated inclusion and exclusion criteria.

### 2.2. Measures

*Socio-demographics.* Socio-demographic variables consisted of age (12–14 years old, 15–17 years old), gender (male and female), ethnicity (Hispanic and non-Hispanic), race (White only, Black only, and others), and annual household income. Income was collapsed into the following categories: Less than USD 10000, USD 10000–USD 24999, USD 25000–USD 49999, USD 50000–USD 99999, and USD 100000 or more. 

*Outcome Measure.* The outcome measure of interest was the self-reported response from Wave 5 concerning dental health issues diagnosed in the past year. Specifically, it asked the question: In the past 12 months, have you ever been told by a doctor, dentist, or another health professional that you have dental health issues? (Yes/No).

*Familial variables*. Five parental dichotomous variables related to ENDS use were assessed at baseline (Wave 3): (1) In the past 12 months, have your parents or guardians talked with you, even once, about not using ENDS? (Yes/No); (2) Do you think any tobacco products or ENDS might be available to youths at their parent or guardian’s home? (Yes/No); (3) Rules about using ENDS inside the home (Not allowed at all/Allowed or to some extent allowed); (4) Have close biological relatives ever been troubled by SUD? (Yes/No); and (5) If your parents or guardians found you using ENDS, how do you think they would react? (Be very upset/Have no reaction or not be so upset). Table A1 displays the PATH questions used for this study. 

*Risk factors*. Alcohol use, drug use, and tobacco use, all of which are considered risk factors for poor oral health [37]. The alcohol use and drug use variables were dichotomous (Yes/No). The tobacco use variable was coded “Yes” if respondents used at least one of the following: cigarette, cigar, pipe, hookah, snus, smokeless tobacco, dissolvable tobacco, bidi, and kretek; otherwise, it was coded as “No”. Table A2 lists the risk factors.

### 2.3. Statistical Analysis

Descriptive statistics of participants’ baseline socio-demographics, familial factors, and risk factors were calculated using Binary logistic regression models to assess associations between ENDS-related familial factors at baseline (Wave 3) and oral health outcomes at follow up (Wave 5)—controlling for socio-demographics, alcohol use, and tobacco use. Sensitivity analysis was conducted by examining recent use (within the past 12 months) and lifetime use (ever use) of alcohol and tobacco. Variables that had less than 5% of cases in any single response category were not included in regression analyses due to a sparse data matrix. To obtain nationally representative estimates, the Wave 5 ‘all-wave’ weights were applied to the sample estimates. Adjusted odds ratios (AOR) and 95% confidence intervals (95% CI) were reported. All analyses were conducted with IBM SPSS Statistics V28 software, and statistical significance was set at *p* < 0.05.

## 3. Results

Table 1 presents the baseline characteristics of the 3892 adolescents (Weighted N = 22,689,793) aged 12 to 17 years old. Among the respondents, 98% were 12 to 14 years old, and 2% were 15 to 17 years old. Males comprised slightly more than half of the population, and Non-Hispanic and Whites represented the largest Ethnicity/Race group. Most did not have a history of tobacco, alcohol, or substance use. Households with an annual income of USD 100,000 or more were the most common (26.2%). Across the youth population, less than half (43.8%) reported having a parental discussion about not using ENDS, 89% reported no ENDS availability at home, 84.2% reported they were not allowed to use ENDS at home, 74.8% reported having no close biological relatives troubled by a substance use disorder (SUD), and 94.9% reported parents having an extreme reaction (very upset) if they found adolescents using ENDS.

Adolescents with a household income of less than USD 10000 had a higher likelihood of developing dental health issues than those households earning greater than USD 100000 per year (Table 2). After controlling for socio-demographic characteristics and lifetime use of alcohol and tobacco, any tobacco or ENDS availability at home (AOR = 1.132, 95% CI = 0.578–2.218), and having biological relatives who have ever been troubled by SUD (AOR = 1.301, 95% CI = 0.818–2.070), were not associated with oral health (Table 2). Lack of in-house rules (AOR = 01.200, 95% CI = 0.616–2.342) and parental discussions about not using ENDS (AOR = 0.710, 95% CI = 0.461–1.093) were also not linked to dental health issues (Table 2). However, parents’ extremely upset reaction when finding out their children use ENDS was linked to a lower risk of dental problems (AOR = 0.309, 95% CI = 0.106–0.905; Table 2). These findings are generally consistent with the results obtained after controlling for demographics and the past 12 months’ use of alcohol and tobacco (Table A3).

## 4. Discussion

This study used a nationally representative PATH database to examine the impact of ENDS-related familial factors on the oral health of adolescents in the United States. It represents the first study examining what familial factors, if any, related to ENDS use impact an adolescent’s oral health. The results indicate that the lack of a negative parental reaction if found using ENDS emerged as an independent predictor for poorer oral health. 

The findings reported here suggest that addressing familial factors offers an opportunity to improve the oral health of adolescents. Good oral health depends on the dynamic interactions between an individual and their cultural, psychological, social, economic, and political environment. For children and teens, health depends on a strong family environment. Our findings indicate that opportunities may exist to strengthen the familial environment in ways that support oral health at both the provider level and in the public health domain.

Cigarette smoking significantly increases the risk of periodontal disease [38,39,40]. Similarly, ENDS use can also be linked to poorer oral health, and the social history that oral health providers obtain should include asking teens about their smoking habits—including ENDS use. Even though social history is an important part of a comprehensive oral health assessment [41], dentists typically place less emphasis on social history taking [42]. This lack of emphasis by dentists on taking and reviewing a patient’s social history may stem from their dental school education, where students focus on completing clinical procedures as a pathway to graduation. Our findings indicate that in addition to traditional social history questions related to alcohol, smoking, and substance use, dentists have an opportunity to improve a patient’s overall health by assessing familial factors related to ENDS use. Although dentists may find it challenging to ask these questions and address these factors, behavior theory suggests that interventions by dentists can alter health behaviors [43]. Since parental reactions related to using ENDS is associated with oral health, advising parents to respond strongly to oppose ENDS use seems likely to be effective. In contrast, setting rules about ENDS use within the past 12 months did not reduce the risk, implying that this might be a less effective strategy. Similar to smoking, where parental attitudes predict use [44] and a parent’s dental habits influence their children’s oral health [45], parental attitudes to ENDS use also predict oral health.

Our study found that those with a household income of less than USD 10,000 were more likely than those households earning greater than USD 100,000 per year to report dental problems, but this may be due to higher income earners’ ability to pay for comprehensive dental care, whereas those with low incomes may delay visiting a dentist until they experience dental problems [46]. Further research is needed to unravel whether these factors represent markers of awareness of a dental issue or true associations with disease.

The results also suggest that public health measures addressing familial factors offer an opportunity to improve oral health. Public health dentistry focuses on the community rather than the individual to prevent and control dental disease [47]. Progress in public health dentistry depends on finding underlying causes that contribute to poor oral health, and then designing, testing, and evaluating interventions to improve health outcomes. This study identified familial factors as a social issue associated with poor oral health, and research that plans, implements, and evaluates strategies to address familial factors offers the potential to improve the oral health of adolescents. Instead of operating at the level of the patient/provider, public health uses a community approach and incorporates public health agencies, private organizations, public policymakers, educators, and other stakeholders with an interest in oral health. Since oral health is an important contributor to an individual’s general health, interventions aimed at addressing familial factors can contribute to the overall health of the nation. 

### Strengths and Limitations

There are several limitations to this study. First, the data were self-reported, and participants might provide socially desirable responses rather than their true responses. Another limitation is that the last data collection occurred in 2019, and recent legislative changes and publicity about adverse ENDS effects might yield different results if more recent data were available. A third limitation is that the PATH survey contains limited response options, but more detailed response options could provide better insight.

In contrast, a study strength is that it used a database representative of the United States youth population. In addition, assessing longitudinal data allowed the selection of youths without dental problems at baseline, to then identify if over time the presence of ENDS-related familial factors predicted future oral health.

## 5. Conclusions

This study used the PATH database to examine the longitudinal effect of ENDS-related parental factors on oral health and found that the lack of a negative parental reaction if found using ENDS adversely affected oral health in adolescents and emerged as an independent risk factor for poor oral health. These findings suggest that strengthening the family environment at both the provider level and in the public health domain may improve the oral health of adolescents.

## Figures and Tables

**Table 1:** Participant characteristics at baseline (Wave 3, PATH 2015–2016).

Variable	Unweighted n (%)	Weighted N (%)	
Age group			
12 to 14 years old	3811 (97.9%)	22,235,420 (98%)	
15 to 17 years old	81 (2.1%)	454,373 (2%)	
Gender			
Male	2001 (51.6%)	11,834,042 (52.4%)	
Female	1878 (48.4%)	10,766,988 (47.6%)	
Ethnicity			
Hispanic	1078 (29.0%)	6,293,253 (28.9%)	
Non-Hispanic	2643 (71.0%)	15,451,559 (71.1%)	
Race			
White alone	2480 (67.4%)	14,563,023 (67.7%)	
Black alone	605 (16.4%)	3,499,123 (16.3%)	
Other	594 (16.1%)	3,439,887 (16.0%)	
Annual household income			
Less than USD 10,000	313 (8.6%)	1,844,438 (8.7%)	
USD 10,000 to USD 24,999	627 (17.2%)	3,743,106(17.7%)	
USD 25,000 to USD 49,999	821 (22.6%)	4,677,549 (22.1%)	
USD 50,000 to USD 99,999	916 (25.2%)	5,342,888 (25.3%)	
USD 100,000 or more	958 (26.4%)	5,540,257 (26.2%)	
Ever used tobacco products?			
No	3492 (94.0%)	20,328,574 (93.8%)	
Yes	222 (6.0%)	1,333,450 (6.2%)	
Ever used alcohol at all?			
No	1074 (86.1%)	6,289,985 (86.5%)	
Yes	173 (13.9%)	980,820 (13.5%)	
Ever used drugs?			
No	1244 (99.8%)	7,241,589 (99.8%)	
Yes	3 (0.2%)	15,230 (0.2%)	
In past 12 months, used tobacco products?			
No	2499 (96.2%)	14,546,784 (96.0%)	
Yes	100 (3.8%)	605,606 (4.0%)	
In past 12 months, used alcohol at all?			
No	2181 (83.6%)	12,780,447 (84.1%)	
Yes	428 (16.4%)	2,421,499 (15.9%)	
In past 12 months, used drugs?			
No	2574 (99.2)	14,975,729 (99.1%)	
Yes	21 (0.8)	134,884 (0.9%)	
In the past 12 months, have your parents or guardians talked with you, even once, about not using ENDS?			
No	2140 (56.1%)	12,519,245 (56.2%)	
Yes	1675 (43.9%)	9,743,059 (43.8%)	
Do you think any tobacco products or ENDS might be available to youths at parent or guardian’s home?			
No	3458 (88.8%)	20,188,911 (89.0%)	
Yes	434 (11.2%)	2,500,882 (11.0%)	
Rules about using ENDS inside the home			
Not allowed at all	3194 (84.1%)	18,634,653 (84.2%)	
Allowed in some extent allow Allowed anywhere at anytime	338 (8.9%) 265 (7.0%)	1,945,282 (8.8%) 1,557,314 (7.0%)	
Have close biological relatives ever been troubled by SUD?			
No	2893 (75%)	16,804,842 (74.8%)	
Yes	965 (25%)	5,673,385 (25.2%)	
If your parents or guardians found you using ENDS, how do you think they would react?			
Be very upset	3636 (94.8%)	21,221,840 (94.9%)	
Not be so upset Have no reaction	128 (3.3%) 73 (1.9%)	726,540 (3.3%) 404,938 (1.8%)	

**Table 2:** Effects of ENDS-related familial factors on oral health among youth who had ever used alcohol and tobacco in their life.

	Parents Talking about Not Using ENDS (Ref: No)			Any Tobacco or ENDS Availability at Home (Ref: No)			In-House Rules toward Using ENDS (Ref: Not Allowed at All)			Close Biological Relatives Have Ever Been Troubled by SUD (Ref: No)			Parent Perceived to Have Extreme Reaction If Found Using ENDS (Ref: Have No Reaction)		
	**Past 12 Months Dental Issues at Follow Up**														
**Correlates at baseline**	AOR	95% CI	*p*-value	AOR	95% CI	*p*-value	AOR	95% CI	*p*-value	AOR	95% CI	*p*-value	AOR	95% CI	*p*-value
Familial Factor	0.710	(0.461–1.093)	0.120	1.132	(0.578–2.218)	0.717	1.200	(0.616–2.342)	0.661	1.301	(0.818–2.070)	0.266	0.309	(0.106–0.905)	**0.032**
**DEMOGRAPHIC CHARACTERISTICS**															
**Sex**															
Male	0.708	(0.460–1.088)	0.115	0.707	(0.463–1.081)	0.109	0.709	(0.463–1.086)	0.114	0.706	(0.462–1.079)	0.107	0.651	(0.423–1.003)	0.052
Female	ref	ref	ref	ref	ref	ref	ref	ref	ref	ref	ref	ref	ref	ref	ref
**Ethnicity**															
Hispanic	0.857	(0.493–1.491)	0.585	0.902	(0.520–1.565)	0.715	0.927	(0.533–1.614)	0.789	0.899	(0.524–1.544)	0.700	0.894	(0.511–1.564)	0.695
Non-Hispanic	ref	ref	ref	ref	ref	ref	ref	ref	ref	ref	ref	ref	ref	ref	ref
**Race**															
White alone	0.814	(0.470–1.411)	0.463	0.831	(0.482–1.435)	0.507	0.823	(0.476–1.423)	0.486	0.796	(0.458–1.382)	0.417	0.878	(0.498–1.550)	0.654
Black alone	0.569	(0.262–1.236)	0.154	0.564	(0.260–1.221)	0.146	0.523	(0.237–1.154)	0.108	0.546	(0.252–1.182)	0.124	0.588	(0.268–1.293)	0.186
Other	ref	ref	ref	ref	ref	ref	ref	ref	ref	ref	ref	ref	ref	ref	ref
**Annual household income**															
Less than USD 10,000	2.944	(1.318–6.575)	**0.008**	3.079	(1.372–6.909)	**0.006**	2.769	(1.212–6.325)	**0.016**	2.912	(1.312–6.462)	**0.009**	2.431	(1.103–5.359)	**0.028**
USD 10,000 to USD 24,999	1.884	(0.890–3.985)	0.098	1.843	(0.860–3.950)	0.116	1.848	(0.863–3.956)	0.114	1.734	(0.824–3.649)	0.147	1.820	(0.860–3.852)	0.117
USD 25,000 to USD 49,999	1.622	(0.827–3.181)	0.159	1.701	(0.870–3.326)	0.121	1.681	(0.855–3.305)	0.132	1.619	(0.835–3.318)	0.154	1.580	(0.801–3.116)	0.186
USD 50,000 to USD 99,999	1.024	(.516–2.033)	0.946	1.038	(0.522–2.065)	0.916	1.038	(0.522–2.065)	0.916	0.985	(0.495–1.958)	0.965	1.006	(0.503–2.012)	0.985
USD 100,000 or more	ref	ref	ref	ref	ref	ref	ref	ref	ref	ref	ref	ref	ref	ref	ref
**Ever used tobacco products?**															
Yes	1.406	(0.480–4.119)	0.534	1.364	(0.451–4.124)	0.582	1.473	(0.509–4.261)	0.474	1.267	(0.438–3.670)	0.662	0.756	(0.262–2.183)	0.605
No	ref	ref	ref	ref	ref	ref	ref	ref	ref	ref	ref	ref	ref	ref	ref
**Ever used alcohol at all?**															
Yes	0.684	(0.331–1.412)	0.304	0.643	(0.310–1.331)	0.234	0.651	(0.315–1.344)	0.245	0.651	(0.314–1.349)	0.248	0.622	(0.293–1.320)	0.216
No	ref	ref	ref	ref	ref	ref	ref	ref	ref	ref	ref	ref	ref	ref	ref

Note: Bolded numbers represent *p* < 0.05.

## Data Availability

Data are available at https://www.icpsr.umich.edu/web/NAHDAP/studies/36498/datadocumentation (accessed on 16 January 2022).

## Appendix A

**Table A1:** PATH survey questions and response options related to familial factors.

Question	Response			Recorded Response	
Familial factors					
1. In the past 12 months, have your parents or guardians talked with you, even once, about not using ENDS?	□ Yes	□ No		□ Yes	□ No
2. Do you think any tobacco products or ENDS might be available to youth at parent or guardian’s home?	□ Yes	□ No		□ Yes	□ No
3. Rules about using ENDS inside home	□ It is not allowed	□ It is allowed in some places	□ It is allowed anywhere	□ Not allowed at all	□ Allowed or to some extent allowed
4. Parents reaction if they found out you used e-cigarettes or other electronic nicotine products	□ Be very upset	□ Not be too upset	□ Have no reaction	□ Be very upset	□ Have no reaction or not be too upset
5. Substance use disorders					
Youth’s close biological relatives have ever been an alcoholic or problem drinker	□ Yes	□ No		Youth’s close biological relatives have ever had problems with alcohol or drugs?	
Youth’s close biological relatives have ever had problems with drugs	□ Yes	□ No		□ Yes	□ No

**Table A2:** PATH survey questions and response options related to covariates.

Question	Response		Recoded Response		
**Alcohol use**					
1. In the past 12 months, have you used alcohol at all, including small sips or tastes?	□ Yes	□ No	□ Yes		□ No
2. Have you ever used alcohol at all, including small sips or tastes?	□ Yes	□ No	□ Yes		□ No
**Substance use**					
3. In the past 12 months, have you used any of the following substances? cocaine or crack, stimulants like methamphetamine or speed, any other drugs like heroin, inhalants, solvents, or hallucinogens	□ Yes	□ No	□ Yes		□ No
4. Have you ever used any of the following substances? cocaine or crack, stimulants like methamphetamine or speed, any other drugs like heroin, inhalants, solvents, or hallucinogens	□ Yes	□ No	□ Yes		□ No
**Tobacco Use**					
5. In the past 12 months, have you tried cigarette smoking, even one or two puffs?	□ Yes	□ No	In the past 12 months, have you used any tobacco products?		
6. In the past 12 months, have you smoked a traditional cigar, cigarillo or filtered cigar, even one or two puffs?	□ Yes	□ No			
7. In the past 12 months, have you smoked a pipe filled with tobacco, even one or two puffs?	□ Yes	□ No			
8. In the past 12 months, have you smoked tobacco in a hookah, even one or two puffs?	□ Yes	□ No	□ Yes	□ No	
9. In the past 12 months, have you used snus pouches, even one or two times?	□ Yes	□ No			
10. In the past 12 months, have you used smokeless tobacco products, even one or two times?	□ Yes	□ No			
11. In the past 12 months, have you used dissolvable tobacco products, even one or two times?	□ Yes	□ No			
12. In the past 12 months, have you tried bidis, even one or two times?	□ Yes	□ No			
13. In the past 12 months, have you tried kreteks, even one or two times?	□ Yes	□ No			
14. Have you ever smoked a cigarette, even one or two puffs?	□ Yes	□ No	Have you ever used any tobacco products?		
15. Have you ever smoked a traditional cigar, cigarillo or filtered cigar, even one or two puffs?	□ Yes	□ No			
16. Have you ever smoked a pipe filled with tobacco, even one or two puffs?	□ Yes	□ No			
17. Have you ever smoked tobacco in a hookah, even one or two puffs?	□ Yes	□ No	□ Yes	□ No	
18. Have you ever used snus pouches, even one or two times?	□ Yes	□ No			
19. Have you ever used smokeless tobacco products, even one or two times?	□ Yes	□ No			
20. Have you ever used dissolvable tobacco products, such as Ariva, Stonewall, or Camel Orbs, Sticks, or Strips, even one or two times?	□ Yes	□ No			
21. Have you ever tried bidis, even one or two times?	□ Yes	□ No			
22. Have you ever tried kreteks, even one or two times?	□ Yes	□ No			

**Table A3:** Effects of ENDS-related familial factors on oral health among youth who had used alcohol and tobacco in the past 12 months.

	Parents Talking about Not Using ENDS (Ref: No)			Any Tobacco or ENDS Availability at Home (Ref: No)			In-House Rules toward Using ENDS (Ref: Not Allowed at All)			Close Biological Relatives Have Ever Been Troubled by SUD (Ref: No)			Parent Perceived to Have No Reaction or Not Be So Upset If Found Using ENDS (Ref: Have No Reactiont)		
	**Past 12 Month Dental Issues at Follow Up**														
**Correlates at baseline**	AOR	95% CI	*p*-value	AOR	95% CI	*p*-value	AOR	95% CI	*p*-value	AOR	95% CI	*p*-value	AOR	95% CI	*p*-value
Familial Factor	0.953	(0.719–1.264)	0.740	1.090	(0.717–1.658)	0.686	0.885	(0.535–1.463)	0.827	1.221	(0.909–1.641)	0.184	1.942	(0.509–7.413)	0.332
**DEMOGRAPHIC CHARACTERISTICS**															
**Sex**															
Male	0.999	(0.761–1.312)	0.996	1.004	(0765–1.318)	0.976	1.000	(0.762–1.312)	0.999	1.006	(0.766–1.322)	0.964	1.002	(0.763–1.317)	0.987
Female	ref	ref	ref	ref	ref	ref	ref	ref	ref	ref	ref	ref	ref	ref	ref
**Ethnicity**															
Hispanic	0.919	(0.645–1.308)	0.637	0.955	(0.672–1.358)	0.796	0.927	(0.651–1.320)	0.673	0.987	(0.695–1.402)	0.940	0.972	(0.684–1.380)	0.872
Non-Hispanic	ref	ref	ref	ref	ref	ref	ref	ref	ref	ref	ref	ref	ref	ref	ref
**Race**															
White alone	0.962	(0.661–1.400)	0.840	0.987	(0.679–1.433)	0.945	0.983	(0.678–1.430)	0.936	0.979	(0.671–1.428)	0.911	0.959	(0.661–1.391)	0.826
Black alone	0.661	(0.400–1.092)	0.106	0.682	(0.413–1.127)	0.135	0.683	(0.415–1.126)	0.135	0.671	(0.404–1.113)	0.122	0.682	(0.413–1.125)	0.134
Other	ref	ref	ref	ref	ref	ref	ref	ref	ref	ref	ref	ref	ref	ref	ref
**Annual household income**															
Less than USD 10,000	1.125	(0.600–2.109)	0.713	1.117	(0.549–2.100)	0731	1.138	(0.601–2.156)	0.691	1.078	(0.576–2.017)	0.815	1.139	(0.605–2.143)	0.687
USD 10,000 to USD 24,999	1.773	(1.128–2.788)	**0.013**	1.788	(1.134–2.819)	**0.012**	1.868	(1.186–2.941)	**0.007**	1.689	(1.075–2.653)	**0.023**	1.795	(1.139–2.828)	**0.012**
USD 25,000 to USD 49,999	1.387	(0.905–2.217)	0.133	1.382	(0.897–2.131)	0.142	1.422	(0.925–2.186)	0.108	1.325	(0.863–2.034)	0.198	1.362	(0.887–2.903)	0.158
USD 50,000 to USD 99,999	1.242	(0.836–1.846)	0.283	1.248	(0.840–1.853)	0.273	1.269	(0.856–1.882)	0.235	1.211	(0.815–1.800)	0.344	1.255	(0.845–1.862)	0.260
USD 100,000 or more	ref	ref	ref	ref	ref	ref	ref	ref	ref	ref	ref	ref	ref	ref	ref
**Used tobacco products in the past 12 months?**															
Yes	1.323	(0.707–2.477)	0.382	1.324	(0.707–2.479)	0.381	1.333	(0.715–2.485)	0.366	1.337	(0.721–2.482)	0.357	1.262	(0.664–2.401)	0.478
No	ref	ref	ref	ref	ref	ref	ref	ref	ref	ref	ref	ref	ref	ref	ref
**Used alcohol in the past 12 months?**															
Yes	0.939	(0.643–1.370)	0.743	0.925	(0.634–1.349)	0.685	0.927	(0.636–1.352)	0.694	0.933	(0.638–1.363)	0.718	0.948	(0.648–1.386)	0.783
No	ref	ref	ref	ref	ref	ref	ref	ref	ref	ref	ref	ref	ref	ref	ref

Note: Bold numbers represent *p* < 0.05.
